# The Comparison of Efficacy of Bilateral Type I Versus Type II Pectoral Nerve Block Applied with Ultrasound-Guided for Postoperative Analgesia in Gynecomastia Surgeries

**DOI:** 10.1007/s00266-025-04865-1

**Published:** 2025-04-23

**Authors:** Aykut Urfalıoglu, Mehmet Bekerecioglu, Adem Doganer, Harun Karaduman, Gizem Ezgi Satıcı, Abdullah Dincgözoglu

**Affiliations:** 1 Department of Anesthesiology and Reanimation, Faculty of Medicine, Kahramanmaras Sutcu Imam University, Avsar Campus, 46100 Onikisubat, Kahramanmaras, Turkey; 2 Department of Plastic Reconstructive and Aesthetic Surgery, Faculty of Medicine, Kahramanmaras Sutcu Imam University, Kahramanmaras, Turkey; 3 Department of Biostatistics and Medical Informatics, Faculty of Medicine, Kahramanmaras Sutcu Imam University, Kahramanmaras, Turkey

**Keywords:** Pectoral nerve (PECS) block, Gynecomastia, Postoperative analgesia

## Abstract

**Background:**

Gynecomastia surgeries are frequently performed due to esthetic concerns. There is lack of data regarding postoperative pain control for these patients. The aim of this study was to compare the pectoral nerve (PECS) block type I and type II with respect to postoperative analgesic consumption, pain scores in these patients.

**Methods:**

The prospective randomized study was conducted with 30 patients, with American Society of Anesthesiologists I–II, between the ages of 18 and 50, and undergoing bilateral gynecomastia surgery. The patients were divided into two groups: PECS I block with general anesthesia (*n* = 15) and PECS II block with general anesthesia (*n* = 15). The demographic data, hemodynamic parameters, postoperative numeric rating scale (NRS) scores (at 0, 1, 2, 6, 12, 24 h postoperative), the number of patients who needed rescue analgesia, and block-related adverse events were recorded.

**Results:**

NRS scores at 30 min, 1 and 2 h postoperatively were similar in the two groups, whereas the scores at 6, 12 and 24 h were significantly lower in the PECS II group (*p* = 0.005, *p* = 0.007, *p* = 0.002, respectively). It was determined that the postoperative 24-h tramadol consumption was statistically significantly lower in the PECS II group (*p* = 0.005). Additional analgesic was required in two patients in the PECS I group, but none in the PECS II group.

**Conclusion:**

PECS blocks could effectively reduce postoperative pain level in gynecomastia operations; however, PECS II block was superior to PECS I block in terms of both analgesic consumption and pain scores.

**Level of Evidence II:**

This journal requires that authors assign a level of evidence to each article. For a full description of these Evidence-Based Medicine ratings, please refer to the Table of Contents or the online Instructions to Authors www.springer.com/00266.

## Introduction

The use of ultrasound-guided thoracic fascial plane blocks such as erector spina plan (ESP) block, serratus anterior plan (SAP) block, pectoral nerve (PECS) block, paravertebral and intercostal blocks as part of multimodal analgesia protocols for postoperative pain control is becoming widespread [1]. Similarly, it is seen in anesthesia practice that these fascial plane blocks are increasingly applied in many esthetic breast surgery procedures [2–6].

Pectoral nerve block type I (PECS I), first described by Blanco, has been reported to be used as an alternative to paravertebral and epidural blocks in pain management after thoracic wall surgical interventions, especially breast surgeries [7]. The nerve targeted in this ultrasound-guided block is the lateral pectoral nerve between the pectoralis major (PMm) and pectoralis minor (Pmm) and the medial pectoral nerve innervating the Pmm. In the modified PECS block, also known as pectoral nerve block type II (PECS II), a type I block is performed by targeting the fascia between PMm and Pmm, and a second block is applied by administering local anesthetic to the fascia between Pmm and Serratus anterior muscle (SAM) further down. Thus, in addition to the medial and lateral pectoral nerves in the type I block, the 3rd-6th intercostal, intercostobrachial and long thoracic nerves are blocked [8]. With PECS II block, it is known that after breast cancer surgeries requiring intervention in deeper tissues, and/or esthetic surgeries such as reduction mammoplasty and breast augmentation, postoperative analgesic efficacy and possible side effects due to less opioid use are reduced [5, 9–11]. Although it has been reported that PECS I block should be preferred mostly in superficial surgeries due to its inability to provide deep nerve blockade [12], there are also studies mentioning its superior postoperative analgesic effects in breast augmentation surgeries [13, 14].

In gynecomastia, which is called an increase in breast tissue in men, surgical intervention may be required due to esthetic concerns, and surgical trauma is relatively minimal compared to breast surgeries performed in women. In these operations, gland tissue hypertrophy on the pectoral muscle, if any, is excised along with liposuction applied to adipose tissue**.** Although surgery is not performed on deep tissues, sometimes surgical trauma to the pectoral muscle fascia may cause severe postoperative pain. Many different methods have been described for pectoral fat extraction during gynecomastia surgery, including traditional, ultrasonic, power-assisted or combined. When we look at the recent developments in the literature, a combined method including both liposuction, fat injection and open resection, called open resection and high-definition liposcalpture, has been described by Hoyos et al. [15].

In light of this information, in this prospective randomized study, we aimed to compare the efficacy of bilateral ultrasonographic PECS I and PECS II blocks applied for postoperative analgesia in gynecomastia operations in terms of postoperative pain scores and total analgesic consumption. To our knowledge, this is the first study in the literature comparing the postoperative analgesic efficacy of PECS I and PECS II blocks in gynecomastia operations.

## Materials and Methods

The prospective randomized study was initiated following the approval of Kahramanmaras Sutcu Imam University Faculty of Medicine Clinical Research Ethics Committee (Decision no: 2022/26-113). After detailed information about the operation and the study, written consent and signatures were obtained from patients between the ages of 18–50 years and ASA I-II according to the ASA (American Society of Anesthesiologists) risk score, who were to be operated for gynecomastia. Patients outside the age range of 18–50 years, with a body mass index (BMI)≥ of 35 kg/m^2^ an ASA III risk score≥ Mallampati score≥ of 3 on preoperative examination, with severe co-morbid diseases (bleeding diathesis, abnormal coagulation tests, anticoagulant use, known local anesthetic allergy/toxicity, previous major trauma/surgery, uncontrolled hypertension, history of cardiac, cerebrovascular, neuromuscular, renal, hepatic diseases, etc.), presence of infection at the site of the block were determined as exclusion criteria.

### Preoperative Preparation

In the preoperative preparation room, 30 patients were randomly assigned to either group PECS I: (*n* = 15, type I pectoral nerve block patients) or group PECS II: (*n* = 15, type II pectoral nerve block patients). All patients were admitted to the operating room after 2 mg intravenous (IV) midazolam premedication. Demographic data (age, BMI, ASA) were recorded and standard monitoring was performed and general anesthesia induction was started after PECS I or PECS II block was performed by a single anesthesiologist (A.U.) according to the study group. Block times between needle entry and the end of the block were recorded in both groups. After the standard anesthesia protocol was applied, in order to reduce bleeding in the surgical field, tumescent solution prepared by adding 1 ml of 0.1% (1/1000) adrenaline in 1000 ml of Ringer's lactate and containing no local anesthetic was given to both breasts approximately 250–300 ml each by the surgeon and then the surgical procedure was started. The time between the administration of tumescent solution and the end of the surgical procedure was recorded as the operation time.

### PECS I and PECS II Block Applications

The block procedure was performed in all patients in the supine position with the arm in 90 degrees of abduction. After sterilization with povidone-iodine, the procedure was performed with a 22-gauge 50 mm (Stimuplex D, Braun, Melsungen, Germany) Quincke type block needle (Stimuplex D, Braun, Melsungen, Germany), following the aseptically coated high-frequency (4-12 MHz) linear probe of the ultrasound device (GE LOGIQ^TM^ eR7 Ultrasound System, General Electric Co, USA) .

**Fig. 1:**
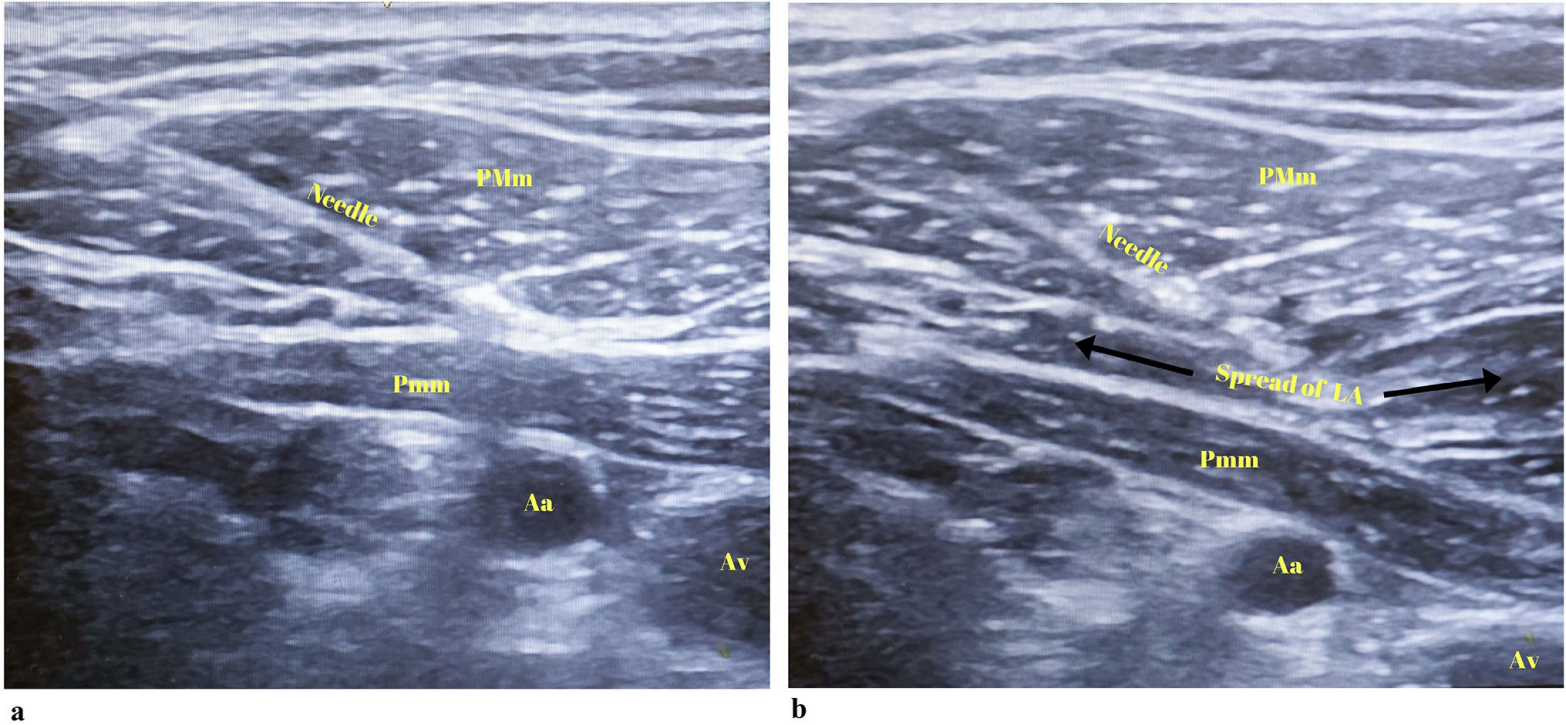
The view of anatomical structures and local anesthetic spread during Pectoral nerve block type I (PECS I) application (PMm: pectoralis major muscle, Pmm: pectoralis minor muscle, Aa: Axillary artery, Av: Axillary vein, LA: local anesthetic). **a** Ultrasound image of needle direction between the muscles in PECS I block. **b** Spread of local anesthetic at the plane in PECS I block

**Fig. 2:**
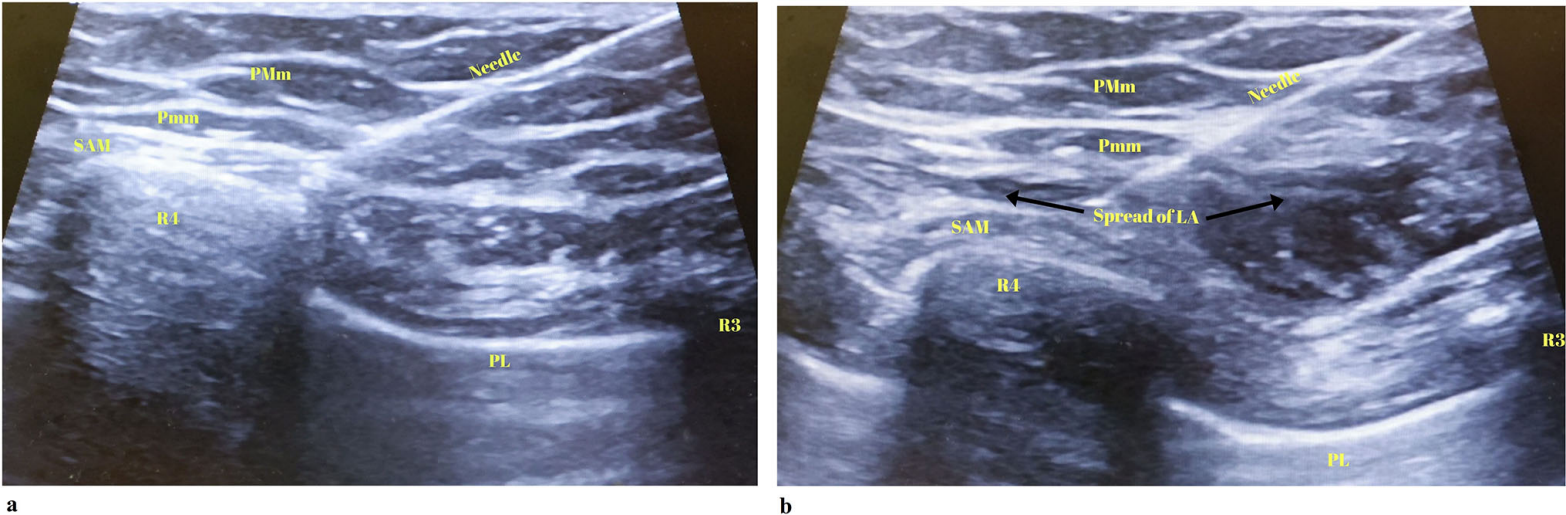
The view of anatomical structures and local anesthetic spread during Pectoral nerve block type II (PECS II) application (PMm: pectoralis major muscle, Pmm: pectoralis minor muscle, SAM: serratus anterior muscle, LA: local anesthetic, R3: third rib; R4: fourth rib, Pl; Pleura). **a** Ultrasound image of needle direction between the muscles in PECS II block. **b** Spread of local anesthetic at the plane in PECS II block

During PECS I block, the linear probe was first placed sagitally between the lateral clavicle and the acromio-clavicular joint to visualize the axillary artery/vein. Lateral rotation of the probe visualized the pectoral branch of the thoracoacromial artery located between the PMm and Pmm muscles on the 2nd rib and the fascia of these muscles. This point was determined as the point where the needle would be inserted. After the needle was inserted at this point, negative aspiration was performed followed by hydrodissection with 0.5–1 ml saline and 20 ml of 0.25% bupivacaine was administered here after the location was confirmed. The same procedure was performed on the other side (Fig. 1). When performing PECS II block, the probe was placed in the same position as PECS I, the thoracoacromial artery and the fascia between the PMm and Pmm muscles were visualized at the level of the 2nd rib and 10 ml of 0.25% bupivacaine was administered and PECS I block was performed first. Then, the probe was rotated to form an angle from the midclavicular line to the inferolataral and first the axillary artery/vein and the 2nd rib were visualized, and then the 3rd and 4th ribs were visualized by moving infereriorly in the same line. At this level, the SAM muscle was visualized below the PMm and Pmm muscles on the 4th rib. After negative aspiration of 0.5–1 ml of saline and hydrodissection of the fascia between Pmm and SAM, 20 ml of 0.25% bupivacaine was administered by confirming the location of the needle. The same procedure was performed on the other side (Fig. 2).

### Standard Anesthesia and Postoperative Follow-up Protocol

Propofol 2–3 mg/kg IV, fentanyl 1.5 mcg/kg IV, and rocuronium 0.6 mg/kg IV as a neuromuscular blocker were administered as standard in patients to undergo general anesthesia and endotracheal intubation was started at the end of the 3rd min. For maintenance of anesthesia, standard 50% O2/50% medical air mixture and 2% sevoflurane inhalation with remifentanil infusion as analgesic and rocuronium IV when necessary were administered during the operation. In the standart monitoring of the patients, heart rate (HR), mean blood pressure (BP) and peripheral oxygen saturation (SpO2) values were recorded at perioperative intervals (T0, T1, T2, T3).

Each patient recieved tramadol HCl 50 mg (Contramal amp, Abdi Ibrahim Pharma., Turkey) IV as a standard analgesic 20 min before the end of surgical intervention. At the end of anesthesia, Sugammadex 2 mg/kg IV was administered to all patients as a neuromuscular blocker antagonist and the patients were extubated after spontaneous respiration to create sufficient tidal volume. The patient-controlled analgesia (PCA) device was attached, and the patient was sent to the recovery room.

### Surgical Technique

All surgical operations were performed by a single surgeon (M.B.) with the same surgical method. Only patients with Webster classification type II (mix fatty and glandular breast tissue) and Simon classification stage-2a (moderate breast hypertrophy/ no excess skin) were included in this study, and the same drawings were made preoperatively for all patients. [16, 17]. While the patient was standing, the inframammary fold (IMF), breast borders and planned incision sites along the lateral IMF were drawn, respectively. Concentric topographic areas that form the most prominent area of the breast and where liposuction is planned to be performed were marked. For all areas to be treated, it was planned to use a 3.0-mm cannula at multiple levels and to aspirate in a 1:1 ratio with super-wet tumescent technique. Two incisions were planned for liposuction, the lateral IMF and the upper anterior axilla, and a standard omega incision for subareolar glandular excision if necessary. The superolateral area on the pectoralis major was marked with a red circle, and liposuction was not performed in this area. The entire IMF line was dissected with a cannula inferiorly. The amount of liposuction obtained from both breasts was recorded [18].

### PCA Protocol

All patients were fitted with a PCA device (APM II Ambulatory Pump, Abbott Laboratories, San Diego, CA, USA), and analgesics were administered by patient preference according to the prepared protocol. Tramadol HCl was adjusted to 5 mg/ml. The device settings were adjusted so that 10 mg (2 ml) tramadol HCl IV was given in each press with only bolus setting without adjusting the infusion, the lockout time was 20 min and the maximum dose to be given in 1 h was 30 mg (6 ml) tramadol HCl IV.

At 30 min (T0) in the recovery room, numeric rating scale (NRS) pain scores [0: no pain,...,10: the most severe pain experienced] of all patients were questioned, and if the scores were 5≥, 50 mg Dexketoprofen (Arveles amp, Pharma., Turkey) was administered IV and transportation to the ward was planned. NRS pain scores of the patients in the ward at 1, 2, 6, 12 and 24 h (T1, T2, T6, T12, T24) were questioned and recorded, and 50 mg dexketoprofen IV was administered as rescue analgesic to patients with scores of 5≥. At the end of 24 h, total tramadol consumption and additional rescue analgesic needs of the patient were questioned and recorded. Postoperative nausea/vomiting and other adverse events (respiratory depression, severe hypotension, hematoma/edema, local anesthetic toxicity, pneumothorax, etc.) were also noted. Ondansetron 4 mg (Zofran amp, Sandoz Pharma., Turkey) was administered IV to patients with nausea/vomiting lasting longer than 10 min. At the 1-month follow-up visits, the surgical team asked the patients whether they had any complaints related to anesthesia, surgery and block procedure during this period and whether they needed reoperation.

### Statistical Analysis

While determining the sample size, G-power analysis was performed using the reference study of Ciftçi et al. [14], and accordingly, considering the VAS 1st h score values, the calculated effect size was 1.38, *α* = 0.05 first type error level and* β* = 0.05 second type error level with a test power of 0.95, and it was determined that the number of samples to be included in each group should be 15 and 30 patients in total.

In the evaluation of the data, the conformity of quantitative variables to normal distribution was examined by Shapiro–Wilk test. Two-group comparisons were made with Independent samples *t* test for variables that conformed to normal distribution and Mann–Whitney *U* test for variables that did not conform to normal distribution. Differences between the frequency distributions of the groups in qualitative variables were analyzed by Chi-square test and Fisher exact test. Statistical parameters were expressed as mean ± standard deviation, median (quartile 25%-quartile 75%), number *(n*) and ratio (%). Statistical significance was accepted as *p* < 0.05. IBM SPSS version 22 (IBM Corparation, Armonk, New York, USA) program was used to evaluate the data.

## Results

**Fig. 3:**
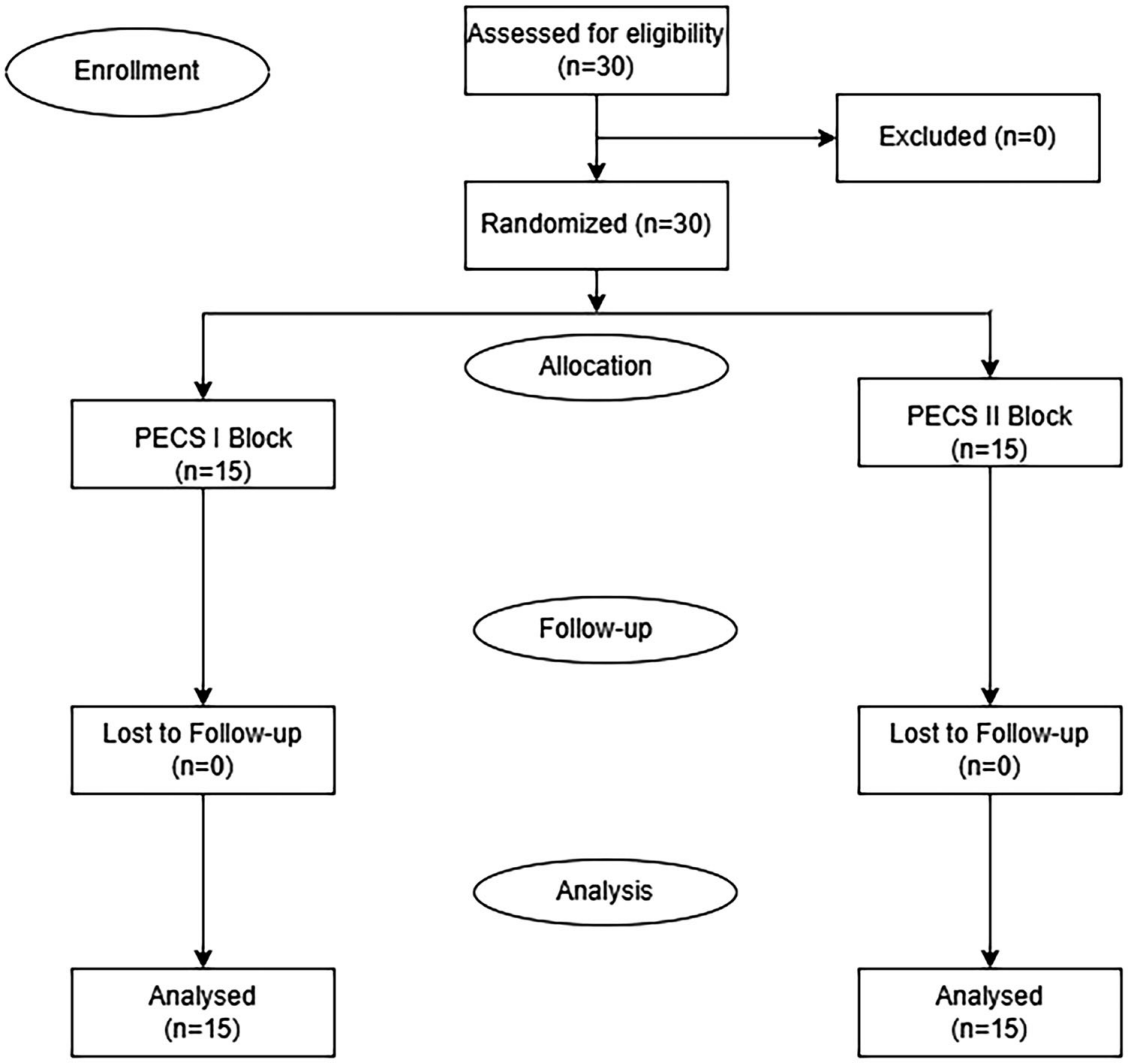
CONSORT flowchart

**Fig. 4:**
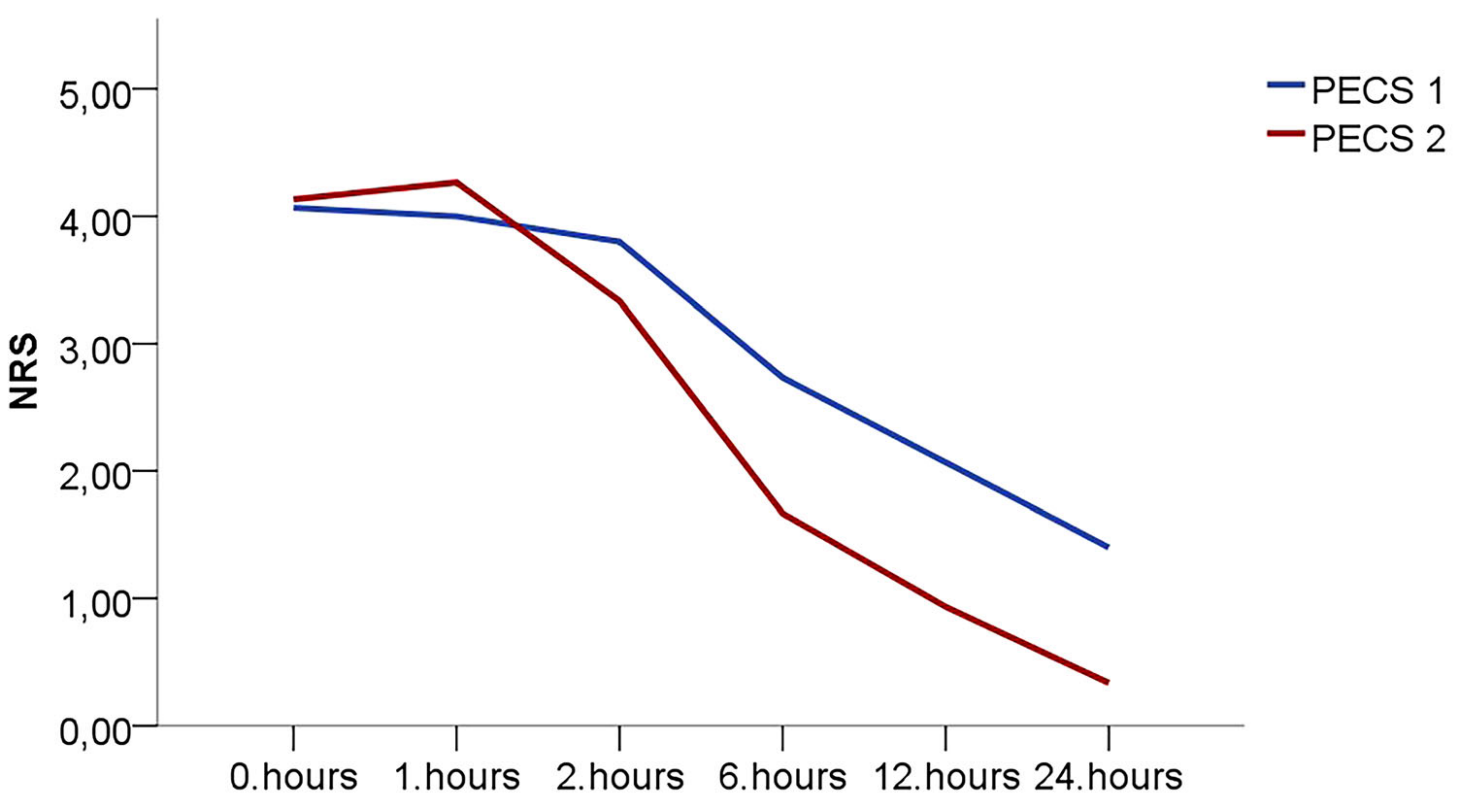
Graphic representation of the postoperative numeric rating scale (NRS) scores in both PECS blocks groups

As seen in the flowchart diagram, among all patients included in the study, no patient was excluded from the study for any reason. Thus, 30 patients who underwent gynecomastia surgery under general anesthesia were randomized and divided into two groups, 15 in PECS I block and 15 in PECS II block (Fig. 3). When demographic and clinical data were analyzed, both groups were similar in terms of age, BMI, ASA scores and operation times. Only the time to perform PECS I block was significantly lower than the time to perform PECS II block (*p* < 0.001) (Table 1). When the hemodynamic data of the patients during the perioperative period were reviewed, it was observed that only the HR value (HR-T2) at the 1st h intraoperatively was higher in the PECS I group (*p* = 0.007). All other hemodynamic parameters were similar in both groups (Table 2). NRS scores, which were considered as the primary outcome of the study, were compared at 30 min and 1, 2, 6, 12 and 24 h postoperatively. The NRS scores at 30 min, 1 and 2 h postoperatively (NRS-T0, NRS-T1, NRS-T2) were similar in two groups, whereas the NRS scores at 6, 12 and 24 h postoperatively (NRS-T6, NRS-T12, NRS-T24) were significantly lower in the PECS II group (*p* = 0.005, *p* = 0.007, *p* = 0.002, respectively) (Table 3) (Fig. 4). Postoperative 24-h tramadol consumption, need for additional analgesics (dexketoprofen), postoperative nausea/vomiting and other complications were evaluated as secondary outcomes. It was determined that postoperative 24-h tramadol consumption measured with the PCA device was statistically significantly lower in the PECS II group (*p* = 0.005). While 2 patients in the PECS I group needed additional analgesic (dexketoprofen), no patient in the PECS II group needed additional analgesic (dexketoprofen), but this did not cause a statistically significant difference between the two groups (*p* = 0.483). When adverse events such as postoperative nausea/vomiting, respiratory depression, severe hypotension, hematoma/edema, local anesthetic toxicity, and pneumothorax were analyzed, it was determined that nausea/vomiting requiring ondansetron use occurred in 1 patient in the PECS I group, but no other adverse event developed in both groups (Table 4). When the records of the 1st month controls were reviewed, it was determined that there were no complaints and/or complications related to anesthesia, surgery and the block performed in the long term.

**Table 1:** Perioperative details of the patients in the PECS block groups

	Groups				
	PECS I	PECS II	ES (95%CI)	Post-hoc power	*p*
Age Median(Q1–Q3)	26.00 (21.00–33.00)	24.00 (21.00–38.00)	0.106		0.770
ASA					
I *n* (%)	7.00 (46.67)	8.00 (53.33)	0.133		0.715
II *n* (%)	8.00 (53.33)	7.00 (46.67)			
BMI (kg/m^2^) (Mean ± SD)	25.99 ± 3.38	26.46 ± 2.23	0.164 (− 0.850 / 1.178)		0.659
Operation Time (min) (Mean ± SD)	79.60 ± 9.05	79.13 ± 11.33	0.046 (− 1.058 / 0.966)		0.902
Block Time (min) Median(Q1–Q3)	9.00 (9.00–11.00)	15.00 (14.00–16.00)	3.254	1.00	< 0.001*
Average total liposuction volume (mL) Median(Q1–Q3)	700 (350–800)	700 (500–700)	0.008		0.983

ASA, American Society of Anesthesiologist Classification; BMI, Body Mass Index; Data are presented as Median (Q1–Q3), Mean ± SD, Independent samples *t* test; Mann–Whitney *U* test; Chi-Square test; *p* < 0.05*: The difference between the groups is significant; ES (Effect Size)

**Table 2:** Comparison of the perioperative hemodynamic parameters between the PECS block groups

	Groups				
	PECS I	PECS II	ES (95% CI)	Post-hoc power	*p*
HR-T0 Median(Q1–Q3)	88.00 (77.00-97.00)	90.00 (78.00-101.00)	0.175		0.632
HR-T1 (Mean ± SD)	93.20 ± 12.60	96.67 ± 11.72	0.285 (-0.732 / 1.302)		0.442
HR-T2 (Mean ± SD)	78.67 ± 10.45	69.00 ± 7.37	1.069 (-2.152 / 0.013)	0.806	0.007*
HR-T3 (Mean ± SD)	91.93 ± 12.55	91.93 ± 10.26	–		1.000
MAP-T0 (Mean ± SD)	98.80 ± 15.98	95.27 ± 8.88	0.297 (-1,29 / 0.734)		0.460
MAP-T1 (Mean ± SD)	99.93 ± 14.87	102.93 ± 13.57	0.381 (-1,402/ 0.641)		0.568
MAP-T2 Median(Q1–Q3)	85.00 (82.00-92.00)	84.00 (75.00-88.00)	0.213		0.227
MAP-T3 (Mean ± SD)	97.13 ± 12.14	95.20 ± 12.52	0.157 (-1.17 / 0.857)		0.671
SpO2-T0 Median(Q1–Q3)	98.00 (98.00-99.00)	99.00 (98.00-99.00)	0.322		0.346
SpO2-T1 Median(Q1–Q3)	99.00 (98.00-99.00)	99.00 (99.00-100.00)	0.458		0.184
SpO2-T2 Median(Q1–Q3)	99.00 (98.00-100.00)	99.00 (99.00-100.00)	0.144		0.676
SpO2-T3 Median(Q1–Q3)	98.00 (98.00-100.00)	99.00 (99.00-100.00)	0.500		0.160

HR, heart rate; MAP, mean arterial pressure; SpO2, oxygen saturation; T0, before anesthesia; T1, after intubation; T2, İntraoperative 1 h; T3, at the end of anesthesia, data are presented as median (Q1-Q3) and mean ± SD, independent samples *t* test; Mann–Whitney *U* test; *p*< 0.05*: The difference between the groups is significant: ES (Effect Size)

**Table 3:** Comparison of the numeric rating scale (NRS) scores across postoperative period between the PECS block groups

	GROUPS				
	PECS I	PECS II	ES (95%CI)	Post-hoc Power	*p*
NRS-T0 Median(Q1–Q3)	4.00 (3.00-5.00)	4.00 (3.00-5.00)	0.008		0.983
NRS-T1 Median(Q1–Q3)	4.00 (3.00-5.00)	4.00 (4.00-5.00)	0.267		0.439
NRS-T2 Median(Q1–Q3)	4.00 (3.00-5.00)	4.00 (3.00-4.00)	0.491		0.164
NRS-T6 Median(Q1–Q3)	3.00 (2.00-3.00)	2.00 (1.00-2.00)	1.131	0.830	0.005*
NRS-T12 Median(Q1–Q3)	2.00 (1.00-3.00)	1.00 (0.00-2.00)	1.075	0.811	0.007*
NRS-T24 Median(Q1–Q3)	1.00 (1.00-2.00)	0.00 (0.00-1.00)	1.250	0.896	0.002*

T0, postoperative 30min; T1, postoperative 1 h; T2, postoperative 2 h; T6, postoperative 6 h; T12, postoperative 12 h; T24, postoperative 24 h; data are presented as Median (Q1-Q3), Mann–Whitney *U* test; *p* < 0.05*: The difference between the groups is significant; ES (Effect Size)

**Table 4:** Comparison of the total tramadol consumption, need for rescue analgesic, between groups and incidence of adverse events

	GROUPS				
	PECS I	PECS II	ES (95%CI)	Post-hoc Power	*p*
Total tramadol consumption (Mean ± SD)	16.20 ± 6,30	9.90 ± 5.20	− 1.091 (− 2.175 / -0.006)	0.822	0.005*
Need for rescue analgesic					
No *n* (%)	13 (86.7)	15 (100.0)			
Yes *n* (%)	2 (13.3)	0 (0.0)	0.267		0.483
Complications					
No *n* (%)	15 (100.0)	15 (100.0)	–		–
Yes *n* (%)	0 (0.0)	0 (0.0)			
Need for Ondansetron					
No *n* (%)	14 (93.3)	15 (100.0)			
Yes *n* (%)	1 (6.7)	0 (0.0)	0.185		1.00

Complications: Respiratory depression, severe hypotension, hematoma/edema, local anesthetic toxicity, pneumothorax, etc. Data are presented as Mean ± SD and %, Independent samples *t* test; Fisher exact test; *p* < 0.05*: The difference between the groups is significant; ES (Effect Size)

## Discussion

In this study, when PECS I and PECS II blocks applied bilaterally under ultrasound guidance for postoperative analgesia in patients undergoing gynecomastia operation under general anesthesia were compared, it was observed that all postoperative pain scores and postoperative total tramadol consumption were significantly lower in the PECS II block group.

Meta-analyses on the application of PECS blocks in esthetic breast surgery in women report that these blocks in combination with multimodal analgesia protocols can provide good postoperative analgesia control. In PECS II blocks, blockade of deep nerves such as intercostal, intercostobrachial and long thoracic nerves significantly decreases postoperative opioid use and pain scores. On the other hand, different results have been reported in studies on the use of PECS I block, in which the more superficial pectoral nerves are blocked, in breast augmentation surgeries [19, 20].When Ciftci et al. applied PECS I block before and after surgery in patients undergoing breast augmentation surgery, they reported an effective postoperative analgesia in both groups, more prominent before surgery [14]. In another study for the same surgical procedure, it was stated that PECS I block application before general anesthesia did not provide effective analgesia compared to placebo. The authors attributed the unsuccessful block to the inability to block the pectoral nerve branches with a single injection and cited the study showing the successful analgesic efficacy of PECS block with triple injection technique in clavicle surgeries as evidence [12, 21]. In addition, it has been stated that breast skin and gland tissues that could not be blocked with PECS I block were the main cause of postoperative pain in breast augmentation surgeries, which was explained by the successful analgesic results of Cooter et al. in breast augmentation surgeries performed only under paravertebral block without general anesthesia [22]. In our study, we compared the two blocks in these aspects in gynecomastia operations, which is one of the esthetic breast surgeries and where less surgical trauma is expected compared to surgeries performed in women. Consistent with the literature, especially in the PECS II block group, low pain scores and a significant decrease in opioid use were observed, which started significantly at the 6th h postoperatively and lasted until the 24th h. Although a relative analgesic effect was also found in PECS I block, this effect was weaker than in PECS II, especially postoperatively. The higher pain scores observed in the first 2 h postoperatively in both blocks were thought to be related to inadequate local anesthetic spread and thus delayed block residence time in the relatively short surgical times despite preoperative administration. Studies comparing PECS II block with ESP block, whose analgesic efficacy has been previously demonstrated after more invasive breast cancer surgeries, reported that PECS II block reduced postoperative pain scores and opioid consumption more than ESP [23–25]. Similarly, it was reported that the high postoperative analgesic efficacy provided by SAP block and intercostal block, which were previously known to provide effective analgesia in thoracic surgeries and were close to PECS II block in terms of application site, could be achieved with PECS II block alone in breast cancer surgery cases [26]. The results of Kim et al. [27] reporting the postoperative analgesic efficacy of PECS II in patients who underwent sentinel lymph node resection in addition to breast cancer surgery are also consistent with our study and prove the efficacy of PECS II block

In paravertebral blocks, which are deeper thoracic blocks applied in breast surgeries, the incidence of serious complications such as pneumothorax, hypotension due to intrathecal spread, respiratory depression, systemic local anesthetic toxicity, and mental status changes has been found to be quite low [28]. Even in large case series in which trunk blocks such as transversus abdominis plan, paravertebral, intercostal, erector spina plan and pectoral nerve blocks were performed for postoperative analgesia in esthetic breast surgeries, all complications were found to be at as low as 1.6% and this rate was explained by the fact that the blocks were performed safely under ultrasound guidance. In the same study, it was reported in the literature that no complications were encountered in any patient who underwent PECS block, and only postoperative nausea and vomiting were observed in %53 as an adverse event [29]. In our study, these theoretically possible complications were not encountered in any patient and no serious intraoperative or postoperative hemodynamic changes were detected. However, when postoperative nausea/vomiting, which may be associated with postoperative analgesic use that may vary depending on the block efficacy, was considered as an adverse event, only one patient complained of nausea/vomiting requiring antiemetic medication. This was interpreted in favor of low postoperative tramadol use after PECS I and PECS II block in accordance with the literature. Similarly, in studies investigating the postoperative efficacy of PECS block in esthetic breast surgeries, postoperative nausea/vomiting rates, which appear to be lower with less use of opioids, support this result [5, 9, 14, 30]. In the study, PECS I and PECS II blocks were performed before general anesthesia. Although sedation was performed beforehand, the fact that the block was performed in awake patients can be considered as a limiting factor that may cause stress. Reducing the general anesthetic exposure of the patients and the possibility that the ultrasonographic image quality may be relatively affected due to the tumescent solution given before surgery and the difficulties to be experienced during the block procedure can be shown as the reason for this choice. Considering that an objective evaluation could not be made due to these patient-related reasons, it was predicted that it would be more appropriate to indirectly evaluate variables such as the onset of block formation and block quality through postoperative NRS scores. The fact that intraoperative analgesic requirement and longer analgesic efficacy after 24 h postoperatively were not evaluated can be stated as other limiting factors. The fact that gynecomastia operations are performed in shorter surgical times compared to breast surgeries performed in women and also the need for a certain time for the onset of the effect of the block due to local anesthetic spread may explain why only postoperative analgesic evaluation was performed rather than intraoperative analgesic evaluation. The limitation of the postoperative analgesic assessment to a 24-h period is also intended to provide a more accurate assessment due to the short hospitalization and discharge times of the patients.

## Conclusion

According to the results of the study, both ultrasound-guided bilateral PECS I and PECS II blocks can effectively reduce postoperative pain level in gynecomastia operations; however, PECS II block was superior to PECS I block in terms of both analgesic consumption and pain scores. Considering the suitability of the equipment and the block experience of the anesthesiologist, PECS block applications as part of a multimodal analgesia protocol may be an effective and safe postoperative analgesia method for patients undergoing gynecomastia surgery. However, it is envisaged that prospective, controlled studies with larger patient series may support these results more strongly.
